# 4,4′-Bis[2-(3,5-dimeth­oxy­phen­yl)ethen­yl]biphen­yl

**DOI:** 10.1107/S1600536811012888

**Published:** 2011-04-13

**Authors:** Alain Collas, Matthias Zeller, Frank Blockhuys

**Affiliations:** a Department of Chemistry, University of Antwerp, Universiteitsplein 1, B-2610 Antwerp, Belgium; b Youngstown State University, One University Plaza, Youngstown, Ohio 44555-3663, USA

## Abstract

The title compound, C_32_H_30_O_4_, crystallizes with three different conformers of the same mol­ecule in the asymmetric unit, which explains the unusually large unit cell volume. The supra­molecular structure is based on inter­actions involving the meth­oxy groups [C⋯O contacts between 3.090 (2) and 3.204 (2) Å, and C—H⋯O contacts between (normalized) 2.40 and 2.71 Å], π–π stacking of the electron-rich meth­oxy-substituted rings [centroid–centroid distances of 3.6454 (9)–3.738 (1) Å] and C—H⋯π contacts (normalized, 2.62–2.97Å).

## Related literature

For related meth­oxy-substituted biphenyls with 4,4′-bis­(2-phenyl­ethen­yl) substitution, see: Vande Velde *et al.* (2002 ▶) [CSD refcode: MODDUE] and Li & Jian (2009 ▶) [CSD refcode: POWYUW]. For a study on the blue-light-emitting properties of a related compound, see: Jin *et al.* (2002 ▶). For the conformations of meth­oxy­benzenes, see: Vande Velde *et al.* (2007 ▶). For the preparation, see: Jin *et al.* (2002 ▶.
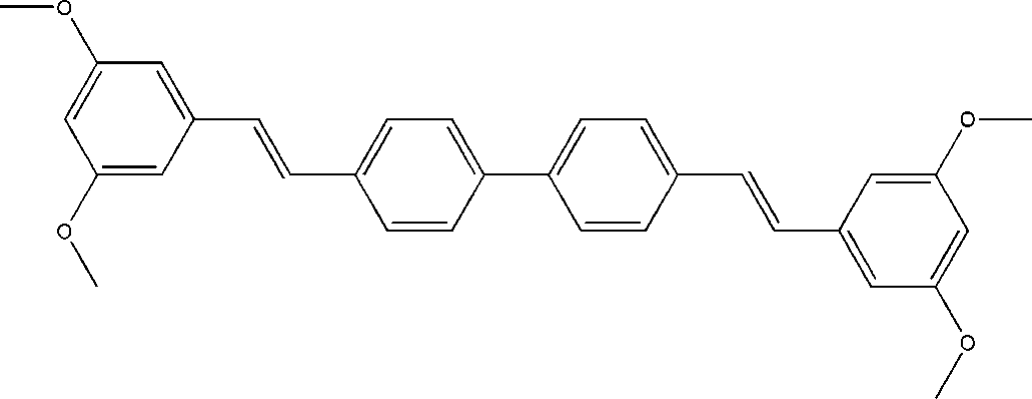


## Experimental

### 

#### Crystal data

C_32_H_30_O_4_
*M* *_r_* = 478.56
Monoclinic, 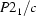

*a* = 11.8208 (13) Å
*b* = 27.896 (3) Å
*c* = 22.875 (3) Å
β = 99.723 (2)°
*V* = 7434.8 (14) Å^3^
*Z* = 12
Mo *K*α radiation
μ = 0.08 mm^−1^
*T* = 100 K
0.55 × 0.45 × 0.15 mm

#### Data collection

Bruker SMART APEX CCD diffractometer
Absorption correction: multi-scan (*SADABS*; Bruker, 2008 ▶) *T*_min_ = 0.956, *T*_max_ = 0.988
74405 measured reflections
18422 independent reflections
13284 reflections with *I* > 2σ(*I*)
*R*_int_ = 0.034

#### Refinement

*R*[*F* ^2^ > 2σ(*F* ^2^)] = 0.060
*wR*(*F* ^2^) = 0.181
*S* = 1.03
18422 reflections
985 parameters
H-atom parameters constrained
Δρ_max_ = 0.68 e Å^−3^
Δρ_min_ = −0.29 e Å^−3^

### 

Data collection: *APEX2* (Bruker, 2008 ▶); cell refinement: *SAINT* (Bruker, 2008 ▶); data reduction: *SAINT*; program(s) used to solve structure: *SHELXS97* (Sheldrick, 2008 ▶); program(s) used to refine structure: *SHELXL97* (Sheldrick, 2008 ▶); molecular graphics: *ORTEP-3* (Farrugia, 1997 ▶) and *Mercury* (Macrae *et al.*, 2008 ▶); software used to prepare material for publication: *WinGX* (Farrugia, 1999 ▶) and *PLATON* (Spek, 2009 ▶).

## Supplementary Material

Crystal structure: contains datablocks I, global. DOI: 10.1107/S1600536811012888/vm2079sup1.cif

Structure factors: contains datablocks I. DOI: 10.1107/S1600536811012888/vm2079Isup2.hkl

Additional supplementary materials:  crystallographic information; 3D view; checkCIF report

## Figures and Tables

**Table 1:** Relevant C—H⋯π contacts in the crystal packing of the title compound (Å, °) *Cg*(*X*1), *Cg*(*X*2), *Cg*(*X*3) and *Cg*(*X*4) are the centroids of the C1*X*–C6*X*, C9*X*–C14*X*, C15*X*–C20*X* and C23*X*–C28*X* rings, respectively, where *X* = *A*, *B*, *C*, *D*.

Entry	*D*	H	*A*	H⋯*A*	*D*—H⋯*A*
1	C10*A*	H10*A*	*Cg*(*B*4)^i^	2.62	148
2	C17*A*	H17*A*	*Cg*(*B*3)^i^	2.86	150
3	C19*A*	H19*A*	*Cg*(*C*4)^ii^	2.80	143
4	C29*A*	H29*A*	*Cg*(*A*3)^iii^	2.79	147
5	C10*B*	H10*B*	*Cg*(*C*1)^iii^	2.84	148
6	C14*B*	H14*B*	*Cg*(*A*2)^iv^	2.97	147
7	C19*B*	H19*B*	*Cg*(*A*1)^iv^	2.67	149
8	C31*B*	H31*F*	*Cg*(*B*2)^iii^	2.80	148
9	C10*C*	H10*C*	*Cg*(*B*1)^iv^	2.70	148
10	C19*C*	H19*C*	*Cg*(*A*4)^v^	2.85	152
11	C29*C*	H29*G*	*Cg*(*C*3)^i^	2.76	143
12	C31*C*	H31*G*	*Cg*(*C*2)^iii^	2.78	140

Symmetry codes: (i) *x* + 1, *y*, *z*; (ii) −*x* + 1, *y* + 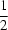, −*z* + 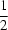; (iii) *x* − 1, *y*, *z*; (iv) *x*, *y*, *z*; (v) −*x* + 2, *y* − 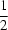, −*z* + 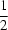.

**Table 2:** Relevant π–π contacts in the crystal packing of the title compound (Å, °) The angle related to a pair of centroids is defined as the angle between the *Cg*(*I*)⋯*Cg*(*J*) vector and the normal to plane *I*. Centroids as in Table 1.

Entry	*Cg*(*I*)	*Cg*(*J*)	*Cg*⋯*Cg*	Angle
1	*Cg*(*A*1)	*Cg*(*A*1)^vi^	3.738 (1)	26.65
2	*Cg*(*A*4)	*Cg*(*A*4)^vii^	3.6454 (9)	25.18
3	*Cg*(*B*1)	*Cg*(*C*4)^viii^	3.713 (1)	25.39
4	*Cg*(*B*4)	*Cg*(*C*1)^ix^	3.697 (1)	26.83
5	*Cg*(*C*1)	*Cg*(*B*4)^*x*^	3.697 (1)	25.86
6	*Cg*(*C*4)	*Cg*(*B*1)^xi^	3.713 (1)	25.72

Symmetry codes: (vi) −*x*, −*y* + 1, −*z*; (vii) −*x* + 3, −*y* + 1, −*z* + 1; (viii) *x* + 1, −*y* + 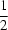, *z* + 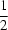; (ix) *x* − 2, −*y* + 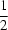, *z* − 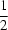; (*x*) *x* + 2, −*y* + 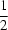, *z* + 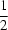; (xi) *x* − 1, −*y* + 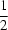, *z* − 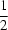.

**Table 3:** Relevant short contacts involving the meth­oxy groups in the crystal packing of the title compound (Å, °)

Entry	*D*	*X*	*A*	*X*⋯*A*	*D*–*X*⋯*A*
1	O2*A*	C30*A*	O1*C*^ix^	3.139 (2)	175.28 (12)
2	O3*A*	C31*A*	O4*C*^*x*^	3.090 (2)	160.33 (12)
3	C32*A*	H32*A*	O2*C*^vii^	2.71	120
4	C32*A*	H32*c*	O3*A*^vii^	2.55	142
5	C15*B*	C16*B*	O1*B*^iii^	3.204 (2)	108.54 (9)
6	O1*B*	C29*B*	O4*A*^vii^	3.171 (2)	143.57 (12)
7	O2*B*	C30*B*	O3*B*^*x*^	3.171 (2)	171.88 (12)
8	O4*B*	C32*B*	O1*A*^xii^	3.102 (2)	174.13 (12)
9	C31*B*	H31*D*	O2*C*^ix^	2.68	139
10	O2*C*	C30*C*	O3*C*^*x*^	3.152 (2)	161.25 (11)
11	C29*C*	H29*H*	O4*B*^*x*^	2.67	141
12	C31*C*	H31*I*	O2*B*^xi^	2.70	143
13	C32*C*	H32*G*	O1*B*^xi^	2.40	144
14	C32*C*	H32*I*	O4*C*^xiii^	2.69	124

Symmetry codes: (xii) −*x* − 1, −*y* + 1, −*z*; (xiii) −*x*, −*y*, −*z*.

**Fig. 1.**
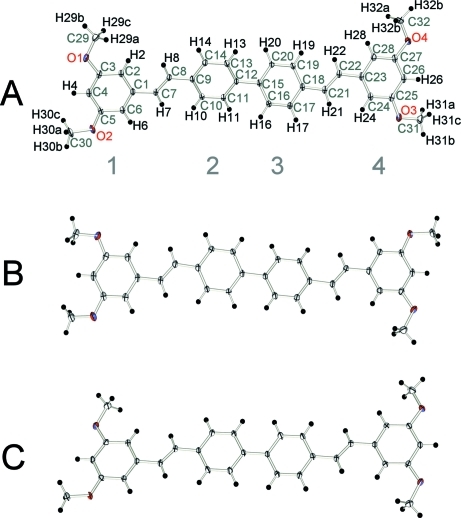
: Molecular structure of the title compound showing the numbering scheme of the three crystallographically independent molecules (A, B, C). Displacement ellipsoids are drawn at the 50% probability level; hydrogen atoms are represented by spheres with an arbitrary radius. Hydrogen atoms of methoxy groups are denominated with a lower case suffix: a, b, c for A, d, e, f for B, and g, h, i for C.

**Fig. 2.**
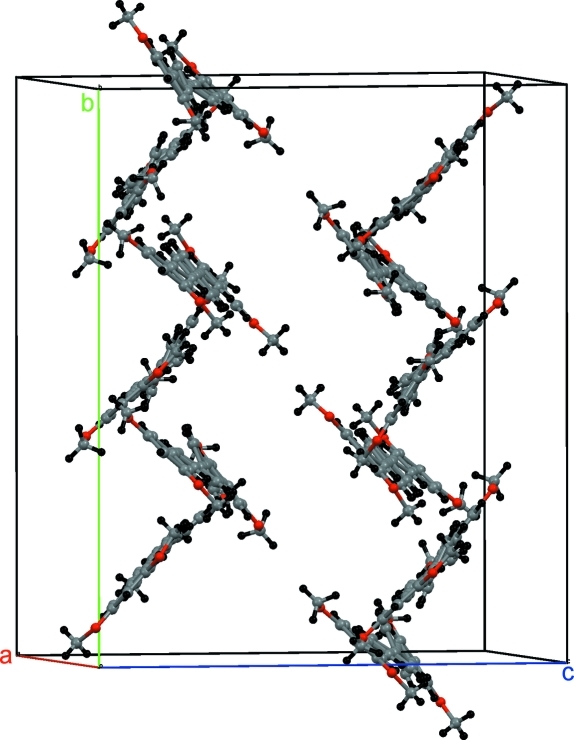
: Packing scheme of the title compound.

## supplementary crystallographic information

### Comment

The title compound was synthesized with its use as the active component in an organic blue-light-emitting diode in mind (Jin *et al.*, 2002). Three conformers of the same molecule are present in the asymmetric unit, displaying different conformations of the methoxy groups and the biphenyl moiety; the molecules have been labeled A, B and C and the numbering scheme is given in Fig. 1. Fig. 2 presents a packing diagram. Molecules A and B have non-planar biphenyl units, with dihedral angles of 30.97 (7)° and 30.51 (7)°, respectively, while the same moiety in molecule C is virtually planar [4.22 (8)°]. In each of the three molecules the methoxy groups are oriented differently: their precise orientations do not have a large influence on the relative stability of the conformer (Vande Velde *et al.*, 2007) but are merely due to the intermolecular contacts they are involved in. The three crystallographically independent molecules are held together by three C–H···π interactions involving aromatic hydrogen atoms. Rings 1 and 2 of molecule A are contacted by H14B and H19B (Table 1, entries 6 and 7) and ring 1 of molecule B is contacted by H10C (Table 1, entry 9). Additionally, molecule A is involved in two methoxy···methoxy contacts (Table 3, entries 1 and 2), two C–H···O contacts involving the methoxy group in the 3-position (Table 3, entries 3 and 4), four C–H···π interactions involving aromatic hydrogen atoms (Table 1, entries 1–4) and two π–π contacts with a symmetry-related A molecule (Table 2, entries 1 and 2). The supramolecular organization of molecule B is based on four methoxy···methoxy contacts (Table 3, entries 5–8), one contact involving the hydrogen atoms of the methoxy group in the 3-position (Table 3, entry 9), two additional C—H···π interactions (Table 1, entries 5 and 8) and two π–π contacts involving the methoxy-substituted rings (1 and 4) of molecules B and C (Table 2, entries 3 and 4). Molecule C participates in five contacts involving the methoxy groups, of which one is a methoxy···methoxy contact (Table 3, entry 10) and four are initiated by hydrogen atoms of the methoxy groups (Table 3, entries 11–14). Two contacts involve the π-systems of the methoxy-substituted rings of molecules B and C (Table 2, entries 5 and 6). Finally, it can be clearly seen from Table 2 that all three molecules are engaged in π–π stacking of the electron-rich methoxy-substituted rings 1 and 4.

### Experimental

The title compound was prepared as is outlined in Yin *et al.* (2002). Crystals suitable for the diffraction experiment were grown by slow evaporation of an acetone solution. *M*.p. (uncorrected) 452 K. ^1^H NMR (CDCl_3_, 400 MHz, TMS): δ 3.83 (s, OCH_3_), 6.41 (t, 2.2 Hz, H4, H26), 6.69 (d, 2.2 Hz, H2, H6, H24, H28), 7.06 (d, 16.3 Hz, H7/H22 or H8/H21), 7.11 (d, 16.3 Hz, H7/H22 or H8/H21), 7.56 (d, 8.4 Hz, H10, H14, H17, H19), 7.61 (d, 8.4 Hz, H11, H13, H17, H19) p.p.m. ^13^C NMR (CDCl_3_, 100 MHz, TMS): δ 55.40 (C29, C30, C31, C32), 100.12 (C4, C26), 104.69 (C2, C6, C24, C28), 127.08 (C10, C14, C17, C19), 127.09 (C11, C13, C16, C20), 128.71 (C7/C22 or C8/C21), 128.81 (C7/C22 or C8/C21), 136.32 (C9, C18), 139.38 (C12, C15), 139.83 (C1, C23), 161.06 (C3, C5, C25, C27) p.p.m.

### Refinement

Hydrogen atoms were placed in calculated positions and refined as riding with C—H distances of 0.93 Å and *U*_iso_(H) = 1.2*U*_eq_(C).

### Crystal data

**Table:**

C_32_H_30_O_4_	*F*(000) = 3048
*M**_r_* = 478.56	*D*_x_ = 1.283 Mg m^−^^3^
Monoclinic, *P*2_1_/*c*	Mo *K*α radiation, λ = 0.71073 Å
Hall symbol: -P 2ybc	Cell parameters from 9749 reflections
*a* = 11.8208 (13) Å	θ = 2.3–32.8°
*b* = 27.896 (3) Å	µ = 0.08 mm^−^^1^
*c* = 22.875 (3) Å	*T* = 100 K
β = 99.723 (2)°	Plate, colourless
*V* = 7434.8 (14) Å^3^	0.55 × 0.45 × 0.15 mm
*Z* = 12	

### Data collection

**Table:**

Bruker SMART APEX CCD diffractometer	18422 independent reflections
Radiation source: fine-focus sealed tube	13284 reflections with *I* > 2σ(*I*)
graphite	*R*_int_ = 0.034
ω scans	θ_max_ = 28.3°, θ_min_ = 1.7°
Absorption correction: multi-scan (*SADABS*; Bruker, 2008)	*h* = −15→15
*T*_min_ = 0.956, *T*_max_ = 0.988	*k* = −37→37
74405 measured reflections	*l* = −30→30

### Refinement

**Table:**

Refinement on *F*^2^	Primary atom site location: structure-invariant direct methods
Least-squares matrix: full	Secondary atom site location: difference Fourier map
*R*[*F*^2^ > 2σ(*F*^2^)] = 0.060	Hydrogen site location: inferred from neighbouring sites
*wR*(*F*^2^) = 0.181	H-atom parameters constrained
*S* = 1.03	*w* = 1/[σ^2^(*F*_o_^2^) + (0.0954*P*)^2^ + 3.3183*P*] where *P* = (*F*_o_^2^ + 2*F*_c_^2^)/3
18422 reflections	(Δ/σ)_max_ = 0.001
985 parameters	Δρ_max_ = 0.68 e Å^−^^3^
0 restraints	Δρ_min_ = −0.29 e Å^−^^3^

### Special details

**Table:**

Geometry. All e.s.d.'s (except the e.s.d. in the dihedral angle between two l.s. planes) are estimated using the full covariance matrix. The cell e.s.d.'s are taken into account individually in the estimation of e.s.d.'s in distances, angles and torsion angles; correlations between e.s.d.'s in cell parameters are only used when they are defined by crystal symmetry. An approximate (isotropic) treatment of cell e.s.d.'s is used for estimating e.s.d.'s involving l.s. planes.
Refinement. Refinement of *F*^2^ against ALL reflections. The weighted *R*-factor *wR* and goodness of fit *S* are based on *F*^2^, conventional *R*-factors *R* are based on *F*, with *F* set to zero for negative *F*^2^. The threshold expression of *F*^2^ > σ(*F*^2^) is used only for calculating *R*-factors(gt) *etc*. and is not relevant to the choice of reflections for refinement. *R*-factors based on *F*^2^ are statistically about twice as large as those based on *F*, and *R*- factors based on ALL data will be even larger. The data has been truncated at 0.75 Å.

### Fractional atomic coordinates and isotropic or equivalent isotropic displacement parameters (Å^2^)

**Table:**

	*x*	*y*	*z*	*U*_iso_*/*U*_eq_	
C1A	0.15447 (12)	0.46499 (5)	0.09560 (6)	0.0168 (3)	
C2A	0.06813 (12)	0.49166 (5)	0.11557 (6)	0.0174 (3)	
H2A	0.0861	0.5124	0.1488	0.021*	
C3A	−0.04359 (12)	0.48741 (5)	0.08623 (7)	0.0181 (3)	
C4A	−0.07196 (12)	0.45719 (5)	0.03724 (7)	0.0188 (3)	
H4A	−0.1490	0.4549	0.0172	0.023*	
C5A	0.01347 (12)	0.43065 (5)	0.01832 (7)	0.0188 (3)	
C6A	0.12663 (12)	0.43462 (5)	0.04730 (7)	0.0193 (3)	
H6A	0.1851	0.4164	0.0339	0.023*	
C7A	0.27507 (12)	0.46747 (5)	0.12364 (7)	0.0180 (3)	
H7A	0.3278	0.4508	0.1040	0.022*	
C8A	0.32024 (12)	0.49024 (5)	0.17333 (6)	0.0155 (3)	
H8A	0.2692	0.5069	0.1941	0.019*	
C9A	0.44267 (12)	0.49152 (5)	0.19832 (6)	0.0147 (3)	
C10A	0.52534 (12)	0.46805 (5)	0.17172 (7)	0.0184 (3)	
H10A	0.5020	0.4505	0.1361	0.022*	
C11A	0.63999 (12)	0.46991 (5)	0.19625 (7)	0.0188 (3)	
H11A	0.6942	0.4537	0.1771	0.023*	
C12A	0.67846 (12)	0.49502 (5)	0.24851 (6)	0.0145 (3)	
C13A	0.59664 (12)	0.51896 (5)	0.27477 (6)	0.0179 (3)	
H13A	0.6202	0.5369	0.3101	0.021*	
C14A	0.48114 (12)	0.51703 (5)	0.25009 (6)	0.0175 (3)	
H14A	0.4270	0.5335	0.2690	0.021*	
C15A	0.80193 (12)	0.49572 (5)	0.27384 (6)	0.0153 (3)	
C16A	0.87267 (13)	0.45705 (6)	0.26583 (7)	0.0223 (3)	
H16A	0.8399	0.4293	0.2455	0.027*	
C17A	0.98942 (13)	0.45819 (6)	0.28679 (7)	0.0236 (3)	
H17A	1.0354	0.4315	0.2801	0.028*	
C18A	1.04088 (12)	0.49784 (5)	0.31748 (6)	0.0175 (3)	
C19A	0.96978 (12)	0.53637 (5)	0.32649 (6)	0.0160 (3)	
H19A	1.0022	0.5638	0.3477	0.019*	
C20A	0.85324 (12)	0.53519 (5)	0.30507 (6)	0.0150 (3)	
H20A	0.8071	0.5619	0.3118	0.018*	
C21A	1.16440 (12)	0.49687 (6)	0.34026 (7)	0.0197 (3)	
H21A	1.2078	0.4722	0.3258	0.024*	
C22A	1.22132 (12)	0.52726 (5)	0.37928 (6)	0.0178 (3)	
H22A	1.1775	0.5521	0.3930	0.021*	
C23A	1.34420 (12)	0.52665 (5)	0.40355 (6)	0.0167 (3)	
C24A	1.41857 (12)	0.49137 (6)	0.39001 (6)	0.0189 (3)	
H24A	1.3902	0.4657	0.3643	0.023*	
C25A	1.53484 (12)	0.49360 (6)	0.41414 (7)	0.0187 (3)	
C26A	1.57761 (12)	0.53051 (5)	0.45167 (6)	0.0184 (3)	
H26A	1.6571	0.5320	0.4678	0.022*	
C27A	1.50255 (12)	0.56549 (5)	0.46552 (6)	0.0180 (3)	
C28A	1.38688 (12)	0.56404 (5)	0.44188 (6)	0.0179 (3)	
H28A	1.3367	0.5883	0.4516	0.021*	
C29A	−0.10905 (14)	0.54661 (7)	0.14674 (7)	0.0280 (4)	
H29A	−0.0678	0.5318	0.1830	0.042*	
H29B	−0.1807	0.5608	0.1548	0.042*	
H29C	−0.0612	0.5716	0.1334	0.042*	
C30A	−0.11888 (14)	0.38859 (7)	−0.05346 (8)	0.0342 (4)	
H30A	−0.1587	0.3759	−0.0225	0.051*	
H30B	−0.1196	0.3645	−0.0847	0.051*	
H30C	−0.1579	0.4177	−0.0704	0.051*	
C31A	1.72111 (13)	0.45930 (7)	0.41990 (8)	0.0301 (4)	
H31A	1.7529	0.4888	0.4060	0.045*	
H31B	1.7582	0.4315	0.4050	0.045*	
H31C	1.7350	0.4588	0.4634	0.045*	
C32A	1.47986 (15)	0.63504 (6)	0.52207 (9)	0.0330 (4)	
H32A	1.4360	0.6513	0.4876	0.050*	
H32B	1.5262	0.6586	0.5474	0.050*	
H32C	1.4269	0.6192	0.5446	0.050*	
C29B	1.22529 (13)	0.35012 (7)	0.42489 (8)	0.0291 (4)	
H29D	1.2407	0.3509	0.4683	0.044*	
H29E	1.2680	0.3759	0.4093	0.044*	
H29F	1.2496	0.3191	0.4110	0.044*	
C2B	0.91825 (13)	0.32978 (6)	0.39548 (7)	0.0194 (3)	
H2B	0.8969	0.3547	0.3675	0.023*	
C3B	1.03183 (13)	0.32419 (6)	0.42179 (7)	0.0199 (3)	
C4B	1.06591 (13)	0.28789 (6)	0.46322 (7)	0.0202 (3)	
H4B	1.1441	0.2844	0.4810	0.024*	
C5B	0.98301 (13)	0.25722 (6)	0.47757 (7)	0.0205 (3)	
C6B	0.86925 (13)	0.26214 (6)	0.45118 (7)	0.0201 (3)	
H6B	0.8135	0.2404	0.4610	0.024*	
C1B	0.83559 (12)	0.29863 (5)	0.41031 (6)	0.0181 (3)	
C7B	0.71374 (12)	0.30156 (6)	0.38449 (6)	0.0186 (3)	
H7B	0.6658	0.2776	0.3968	0.022*	
C8B	0.66159 (12)	0.33367 (5)	0.34585 (7)	0.0192 (3)	
H8B	0.7080	0.3580	0.3330	0.023*	
C9B	0.53876 (12)	0.33459 (5)	0.32160 (6)	0.0176 (3)	
C10B	0.46492 (12)	0.29667 (5)	0.32902 (6)	0.0166 (3)	
H10B	0.4946	0.2691	0.3507	0.020*	
C11B	0.34943 (12)	0.29875 (5)	0.30531 (6)	0.0156 (3)	
H11B	0.3014	0.2724	0.3109	0.019*	
C12B	0.30172 (12)	0.33866 (5)	0.27328 (6)	0.0161 (3)	
C13B	0.37525 (13)	0.37678 (6)	0.26680 (7)	0.0212 (3)	
H13B	0.3452	0.4047	0.2460	0.025*	
C14B	0.49105 (13)	0.37460 (6)	0.29016 (7)	0.0225 (3)	
H14B	0.5391	0.4010	0.2847	0.027*	
C15B	0.17901 (12)	0.34021 (5)	0.24667 (6)	0.0163 (3)	
C16B	0.09571 (12)	0.31668 (5)	0.27237 (7)	0.0189 (3)	
H16B	0.1181	0.2985	0.3075	0.023*	
C17B	−0.01938 (12)	0.31947 (6)	0.24737 (7)	0.0197 (3)	
H17B	−0.0745	0.3032	0.2659	0.024*	
C18B	−0.05605 (12)	0.34550 (5)	0.19575 (7)	0.0170 (3)	
C19B	0.02796 (12)	0.36855 (5)	0.16970 (7)	0.0186 (3)	
H19B	0.0057	0.3863	0.1342	0.022*	
C20B	0.14264 (12)	0.36598 (5)	0.19463 (7)	0.0182 (3)	
H20B	0.1978	0.3821	0.1760	0.022*	
C21B	−0.17861 (12)	0.34744 (5)	0.17100 (7)	0.0184 (3)	
H21B	−0.2294	0.3295	0.1906	0.022*	
C22B	−0.22425 (12)	0.37232 (5)	0.12328 (7)	0.0192 (3)	
H22B	−0.1721	0.3903	0.1047	0.023*	
C23B	−0.34536 (12)	0.37534 (5)	0.09590 (7)	0.0173 (3)	
C24B	−0.43179 (12)	0.34840 (5)	0.11558 (7)	0.0183 (3)	
H24B	−0.4137	0.3275	0.1487	0.022*	
C25B	−0.54361 (12)	0.35257 (5)	0.08623 (7)	0.0177 (3)	
C26B	−0.57203 (12)	0.38340 (5)	0.03787 (7)	0.0174 (3)	
H26B	−0.6490	0.3856	0.0176	0.021*	
C27B	−0.48670 (12)	0.41062 (5)	0.01991 (6)	0.0169 (3)	
C28B	−0.37355 (12)	0.40639 (5)	0.04856 (7)	0.0180 (3)	
H28B	−0.3152	0.4250	0.0355	0.022*	
C30B	1.12008 (15)	0.20950 (7)	0.54112 (8)	0.0331 (4)	
H30D	1.1599	0.1998	0.5087	0.050*	
H30E	1.1228	0.1832	0.5697	0.050*	
H30F	1.1577	0.2378	0.5609	0.050*	
C31B	−0.60962 (15)	0.29225 (6)	0.14510 (8)	0.0283 (4)	
H31D	−0.5607	0.2677	0.1316	0.042*	
H31E	−0.6814	0.2776	0.1523	0.042*	
H31F	−0.5697	0.3067	0.1819	0.042*	
C32B	−0.62030 (14)	0.45357 (7)	−0.04972 (8)	0.0294 (4)	
H32D	−0.6592	0.4247	−0.0674	0.044*	
H32E	−0.6222	0.4784	−0.0802	0.044*	
H32F	−0.6594	0.4653	−0.0180	0.044*	
C6C	1.38454 (12)	0.22574 (5)	0.45759 (6)	0.0158 (3)	
H6C	1.3298	0.2449	0.4729	0.019*	
C5C	1.49943 (12)	0.22673 (5)	0.48485 (6)	0.0160 (3)	
C4C	1.57998 (12)	0.19932 (5)	0.46280 (7)	0.0171 (3)	
H4C	1.6584	0.2001	0.4811	0.021*	
C3C	1.54444 (12)	0.17039 (5)	0.41322 (7)	0.0168 (3)	
C2C	1.43104 (12)	0.16884 (5)	0.38582 (6)	0.0165 (3)	
H2C	1.4083	0.1490	0.3521	0.020*	
C1C	1.34976 (12)	0.19700 (5)	0.40838 (6)	0.0151 (3)	
C7C	1.22788 (12)	0.19699 (5)	0.38199 (6)	0.0164 (3)	
H7C	1.1789	0.2163	0.4011	0.020*	
C8C	1.17803 (12)	0.17304 (5)	0.33432 (6)	0.0167 (3)	
H8C	1.2263	0.1541	0.3143	0.020*	
C9C	1.05527 (12)	0.17327 (5)	0.31000 (6)	0.0151 (3)	
C14C	1.01218 (13)	0.14143 (6)	0.26500 (8)	0.0264 (4)	
H14C	1.0635	0.1204	0.2499	0.032*	
C13C	0.89656 (14)	0.13968 (6)	0.24167 (8)	0.0286 (4)	
H13C	0.8701	0.1172	0.2112	0.034*	
C12C	0.81788 (12)	0.17009 (5)	0.26172 (6)	0.0151 (3)	
C11C	0.86161 (12)	0.20175 (6)	0.30668 (7)	0.0212 (3)	
H11C	0.8104	0.2229	0.3217	0.025*	
C10C	0.97666 (13)	0.20354 (6)	0.33027 (7)	0.0212 (3)	
H10C	1.0029	0.2259	0.3609	0.025*	
C15C	0.69448 (12)	0.16939 (5)	0.23627 (6)	0.0154 (3)	
C20C	0.64884 (13)	0.13538 (6)	0.19423 (7)	0.0231 (3)	
H20C	0.6990	0.1129	0.1808	0.028*	
C19C	0.53336 (13)	0.13335 (6)	0.17159 (7)	0.0238 (3)	
H19C	0.5059	0.1096	0.1430	0.029*	
C18C	0.45600 (12)	0.16546 (5)	0.18980 (6)	0.0162 (3)	
C17C	0.50090 (14)	0.19927 (6)	0.23188 (8)	0.0291 (4)	
H17C	0.4505	0.2214	0.2459	0.035*	
C16C	0.61719 (14)	0.20155 (6)	0.25398 (8)	0.0309 (4)	
H16C	0.6449	0.2257	0.2820	0.037*	
C21C	0.33291 (12)	0.16459 (5)	0.16694 (6)	0.0172 (3)	
H21C	0.2852	0.1838	0.1870	0.021*	
C22C	0.28167 (12)	0.13947 (5)	0.12067 (6)	0.0168 (3)	
H22C	0.3301	0.1205	0.1009	0.020*	
C23C	0.15881 (12)	0.13783 (5)	0.09682 (6)	0.0150 (3)	
C28C	0.12162 (12)	0.10430 (5)	0.05276 (6)	0.0161 (3)	
H28C	0.1754	0.0836	0.0390	0.019*	
C27C	0.00576 (12)	0.10107 (5)	0.02884 (6)	0.0162 (3)	
C26C	−0.07309 (12)	0.13105 (5)	0.04851 (6)	0.0163 (3)	
H26C	−0.1523	0.1287	0.0322	0.020*	
C25C	−0.03562 (12)	0.16461 (5)	0.09225 (6)	0.0162 (3)	
C24C	0.07974 (12)	0.16824 (5)	0.11681 (6)	0.0170 (3)	
H24C	0.1046	0.1912	0.1469	0.020*	
C30C	1.64021 (13)	0.26068 (6)	0.56038 (8)	0.0260 (3)	
H30G	1.6725	0.2292	0.5727	0.039*	
H30H	1.6454	0.2816	0.5951	0.039*	
H30I	1.6834	0.2749	0.5317	0.039*	
C29C	1.59720 (14)	0.10730 (6)	0.35327 (7)	0.0259 (3)	
H29G	1.5527	0.1209	0.3171	0.039*	
H29H	1.5502	0.0838	0.3701	0.039*	
H29I	1.6660	0.0916	0.3438	0.039*	
C32C	0.04090 (15)	0.04176 (6)	−0.04099 (8)	0.0279 (4)	
H32G	0.0907	0.0634	−0.0589	0.042*	
H32H	−0.0014	0.0210	−0.0717	0.042*	
H32I	0.0878	0.0220	−0.0108	0.042*	
C31C	−0.08460 (14)	0.23222 (6)	0.14598 (8)	0.0277 (4)	
H31G	−0.0384	0.2209	0.1831	0.042*	
H31H	−0.1526	0.2491	0.1547	0.042*	
H31I	−0.0387	0.2541	0.1260	0.042*	
O1A	−0.13450 (9)	0.51116 (4)	0.10185 (5)	0.0246 (3)	
O2A	−0.00321 (9)	0.39978 (4)	−0.02838 (5)	0.0269 (3)	
O3A	1.60083 (9)	0.45756 (4)	0.39861 (5)	0.0257 (3)	
O4A	1.55294 (9)	0.60032 (4)	0.50278 (5)	0.0261 (3)	
O1B	1.10602 (9)	0.35647 (4)	0.40456 (5)	0.0259 (3)	
O2B	1.00406 (10)	0.22052 (5)	0.51787 (5)	0.0301 (3)	
O3B	−0.63448 (9)	0.32819 (4)	0.10092 (5)	0.0261 (3)	
O4B	−0.50431 (9)	0.44240 (4)	−0.02580 (5)	0.0232 (2)	
O2C	1.52291 (9)	0.25537 (4)	0.53358 (5)	0.0210 (2)	
O1C	1.63029 (9)	0.14468 (4)	0.39530 (5)	0.0232 (2)	
O4C	−0.03835 (9)	0.06925 (4)	−0.01410 (5)	0.0233 (2)	
O3C	−0.11947 (9)	0.19248 (4)	0.10853 (5)	0.0239 (2)	

### Atomic displacement parameters (Å^2^)

**Table:**

	*U*^11^	*U*^22^	*U*^33^	*U*^12^	*U*^13^	*U*^23^
C1A	0.0112 (6)	0.0184 (7)	0.0195 (7)	−0.0013 (5)	−0.0009 (5)	0.0008 (5)
C2A	0.0129 (7)	0.0200 (7)	0.0181 (7)	−0.0001 (5)	−0.0005 (5)	−0.0009 (6)
C3A	0.0114 (7)	0.0224 (7)	0.0201 (7)	0.0019 (5)	0.0014 (5)	0.0044 (6)
C4A	0.0115 (6)	0.0239 (7)	0.0199 (7)	−0.0029 (5)	−0.0005 (5)	0.0034 (6)
C5A	0.0150 (7)	0.0221 (7)	0.0184 (7)	−0.0056 (6)	−0.0001 (5)	−0.0013 (6)
C6A	0.0124 (7)	0.0210 (7)	0.0241 (8)	−0.0015 (5)	0.0019 (6)	−0.0038 (6)
C7A	0.0108 (6)	0.0191 (7)	0.0228 (7)	0.0006 (5)	−0.0008 (5)	−0.0028 (6)
C8A	0.0105 (6)	0.0164 (7)	0.0188 (7)	0.0002 (5)	0.0005 (5)	0.0020 (5)
C9A	0.0115 (6)	0.0144 (6)	0.0171 (7)	−0.0022 (5)	−0.0009 (5)	0.0024 (5)
C10A	0.0133 (7)	0.0202 (7)	0.0201 (7)	0.0000 (5)	−0.0019 (5)	−0.0056 (6)
C11A	0.0119 (7)	0.0214 (7)	0.0221 (7)	0.0004 (5)	−0.0001 (5)	−0.0059 (6)
C12A	0.0108 (6)	0.0150 (6)	0.0166 (7)	−0.0014 (5)	−0.0012 (5)	0.0007 (5)
C13A	0.0145 (7)	0.0212 (7)	0.0168 (7)	−0.0019 (5)	−0.0006 (5)	−0.0044 (6)
C14A	0.0126 (7)	0.0215 (7)	0.0181 (7)	0.0004 (5)	0.0017 (5)	−0.0017 (6)
C15A	0.0118 (6)	0.0178 (7)	0.0151 (6)	−0.0022 (5)	−0.0015 (5)	−0.0004 (5)
C16A	0.0154 (7)	0.0201 (7)	0.0285 (8)	0.0006 (6)	−0.0046 (6)	−0.0081 (6)
C17A	0.0149 (7)	0.0228 (8)	0.0301 (8)	0.0034 (6)	−0.0048 (6)	−0.0078 (6)
C18A	0.0135 (7)	0.0212 (7)	0.0163 (7)	−0.0018 (5)	−0.0019 (5)	0.0008 (5)
C19A	0.0146 (7)	0.0168 (7)	0.0150 (6)	−0.0033 (5)	−0.0020 (5)	−0.0005 (5)
C20A	0.0131 (7)	0.0159 (7)	0.0150 (6)	−0.0001 (5)	−0.0003 (5)	−0.0001 (5)
C21A	0.0124 (7)	0.0235 (8)	0.0219 (7)	0.0008 (6)	−0.0011 (6)	−0.0007 (6)
C22A	0.0121 (7)	0.0214 (7)	0.0187 (7)	−0.0006 (5)	−0.0007 (5)	0.0023 (6)
C23A	0.0128 (7)	0.0213 (7)	0.0150 (7)	−0.0025 (5)	−0.0005 (5)	0.0049 (5)
C24A	0.0150 (7)	0.0230 (7)	0.0172 (7)	−0.0023 (6)	−0.0014 (5)	0.0012 (6)
C25A	0.0152 (7)	0.0224 (7)	0.0182 (7)	0.0007 (6)	0.0018 (5)	0.0044 (6)
C26A	0.0122 (7)	0.0233 (7)	0.0183 (7)	−0.0035 (5)	−0.0015 (5)	0.0069 (6)
C27A	0.0166 (7)	0.0186 (7)	0.0173 (7)	−0.0044 (5)	−0.0015 (5)	0.0055 (5)
C28A	0.0156 (7)	0.0182 (7)	0.0187 (7)	−0.0008 (5)	−0.0005 (5)	0.0042 (6)
C29A	0.0231 (8)	0.0379 (10)	0.0228 (8)	0.0110 (7)	0.0036 (6)	−0.0032 (7)
C30A	0.0205 (8)	0.0429 (11)	0.0360 (10)	−0.0110 (7)	−0.0046 (7)	−0.0150 (8)
C31A	0.0162 (8)	0.0386 (10)	0.0345 (9)	0.0034 (7)	0.0014 (7)	−0.0026 (8)
C32A	0.0286 (9)	0.0219 (8)	0.0447 (11)	−0.0001 (7)	−0.0052 (8)	−0.0094 (7)
C29B	0.0162 (8)	0.0372 (10)	0.0333 (9)	−0.0027 (7)	0.0027 (7)	−0.0052 (7)
C2B	0.0172 (7)	0.0223 (7)	0.0175 (7)	0.0014 (6)	−0.0007 (6)	−0.0021 (6)
C3B	0.0161 (7)	0.0232 (7)	0.0203 (7)	−0.0014 (6)	0.0028 (6)	−0.0080 (6)
C4B	0.0135 (7)	0.0273 (8)	0.0186 (7)	0.0036 (6)	−0.0006 (5)	−0.0069 (6)
C5B	0.0172 (7)	0.0248 (8)	0.0183 (7)	0.0049 (6)	−0.0001 (6)	−0.0024 (6)
C6B	0.0156 (7)	0.0238 (8)	0.0205 (7)	0.0011 (6)	0.0016 (6)	−0.0021 (6)
C1B	0.0131 (7)	0.0225 (7)	0.0172 (7)	0.0027 (5)	−0.0015 (5)	−0.0045 (6)
C7B	0.0126 (7)	0.0231 (7)	0.0191 (7)	0.0008 (5)	−0.0004 (5)	−0.0023 (6)
C8B	0.0146 (7)	0.0204 (7)	0.0212 (7)	−0.0009 (5)	−0.0013 (6)	−0.0020 (6)
C9B	0.0148 (7)	0.0192 (7)	0.0174 (7)	0.0009 (5)	−0.0017 (5)	−0.0017 (5)
C10B	0.0137 (7)	0.0167 (7)	0.0176 (7)	0.0027 (5)	−0.0023 (5)	0.0004 (5)
C11B	0.0145 (7)	0.0153 (7)	0.0167 (7)	0.0000 (5)	0.0014 (5)	0.0008 (5)
C12B	0.0135 (7)	0.0184 (7)	0.0150 (6)	0.0016 (5)	−0.0016 (5)	−0.0007 (5)
C13B	0.0175 (7)	0.0182 (7)	0.0251 (8)	0.0008 (6)	−0.0043 (6)	0.0042 (6)
C14B	0.0175 (7)	0.0201 (7)	0.0274 (8)	−0.0034 (6)	−0.0032 (6)	0.0032 (6)
C15B	0.0140 (7)	0.0156 (7)	0.0178 (7)	0.0017 (5)	−0.0016 (5)	−0.0006 (5)
C16B	0.0156 (7)	0.0220 (7)	0.0179 (7)	0.0016 (6)	−0.0007 (5)	0.0022 (6)
C17B	0.0147 (7)	0.0229 (8)	0.0210 (7)	−0.0005 (6)	0.0017 (6)	−0.0001 (6)
C18B	0.0123 (7)	0.0166 (7)	0.0207 (7)	0.0018 (5)	−0.0012 (5)	−0.0022 (5)
C19B	0.0145 (7)	0.0189 (7)	0.0206 (7)	0.0025 (5)	−0.0026 (5)	0.0040 (6)
C20B	0.0129 (7)	0.0189 (7)	0.0216 (7)	0.0010 (5)	−0.0005 (5)	0.0047 (6)
C21B	0.0122 (7)	0.0181 (7)	0.0234 (7)	0.0003 (5)	−0.0012 (5)	−0.0022 (6)
C22B	0.0136 (7)	0.0190 (7)	0.0237 (8)	−0.0017 (5)	−0.0006 (6)	0.0004 (6)
C23B	0.0135 (7)	0.0173 (7)	0.0199 (7)	0.0007 (5)	−0.0011 (5)	−0.0032 (6)
C24B	0.0166 (7)	0.0173 (7)	0.0198 (7)	0.0002 (5)	−0.0004 (6)	−0.0005 (6)
C25B	0.0145 (7)	0.0173 (7)	0.0208 (7)	−0.0012 (5)	0.0017 (5)	−0.0033 (6)
C26B	0.0113 (6)	0.0193 (7)	0.0203 (7)	0.0014 (5)	−0.0008 (5)	−0.0048 (6)
C27B	0.0146 (7)	0.0178 (7)	0.0174 (7)	0.0038 (5)	0.0001 (5)	−0.0009 (5)
C28B	0.0123 (7)	0.0177 (7)	0.0233 (7)	−0.0007 (5)	0.0009 (6)	−0.0008 (6)
C30B	0.0234 (9)	0.0391 (10)	0.0352 (10)	0.0069 (7)	−0.0002 (7)	0.0114 (8)
C31B	0.0262 (9)	0.0316 (9)	0.0275 (8)	−0.0060 (7)	0.0061 (7)	0.0034 (7)
C32B	0.0197 (8)	0.0351 (9)	0.0308 (9)	0.0056 (7)	−0.0029 (7)	0.0111 (7)
C6C	0.0117 (6)	0.0150 (6)	0.0200 (7)	0.0003 (5)	0.0005 (5)	−0.0012 (5)
C5C	0.0158 (7)	0.0133 (6)	0.0179 (7)	−0.0030 (5)	−0.0004 (5)	0.0002 (5)
C4C	0.0097 (6)	0.0181 (7)	0.0222 (7)	−0.0017 (5)	−0.0010 (5)	0.0015 (6)
C3C	0.0126 (7)	0.0165 (7)	0.0215 (7)	0.0018 (5)	0.0032 (5)	0.0014 (5)
C2C	0.0132 (7)	0.0173 (7)	0.0181 (7)	−0.0002 (5)	−0.0001 (5)	−0.0012 (5)
C1C	0.0114 (6)	0.0145 (6)	0.0184 (7)	−0.0008 (5)	−0.0001 (5)	0.0009 (5)
C7C	0.0101 (6)	0.0190 (7)	0.0192 (7)	0.0013 (5)	−0.0001 (5)	−0.0014 (5)
C8C	0.0118 (6)	0.0170 (7)	0.0204 (7)	0.0011 (5)	−0.0002 (5)	−0.0014 (5)
C9C	0.0122 (6)	0.0164 (7)	0.0152 (6)	−0.0005 (5)	−0.0019 (5)	0.0002 (5)
C14C	0.0171 (7)	0.0277 (8)	0.0306 (9)	0.0085 (6)	−0.0072 (6)	−0.0138 (7)
C13C	0.0189 (8)	0.0279 (9)	0.0336 (9)	0.0064 (6)	−0.0112 (7)	−0.0177 (7)
C12C	0.0135 (7)	0.0162 (7)	0.0141 (6)	−0.0011 (5)	−0.0022 (5)	−0.0005 (5)
C11C	0.0112 (7)	0.0285 (8)	0.0233 (8)	0.0004 (6)	0.0012 (6)	−0.0118 (6)
C10C	0.0136 (7)	0.0261 (8)	0.0224 (7)	−0.0021 (6)	−0.0012 (6)	−0.0121 (6)
C15C	0.0124 (7)	0.0170 (7)	0.0152 (7)	−0.0008 (5)	−0.0025 (5)	0.0002 (5)
C20C	0.0131 (7)	0.0304 (8)	0.0254 (8)	0.0002 (6)	0.0023 (6)	−0.0146 (6)
C19C	0.0131 (7)	0.0305 (8)	0.0260 (8)	−0.0014 (6)	−0.0014 (6)	−0.0157 (7)
C18C	0.0139 (7)	0.0166 (7)	0.0167 (7)	−0.0010 (5)	−0.0012 (5)	−0.0009 (5)
C17C	0.0162 (8)	0.0297 (9)	0.0366 (9)	0.0094 (6)	−0.0095 (7)	−0.0164 (7)
C16C	0.0201 (8)	0.0278 (9)	0.0386 (10)	0.0064 (6)	−0.0128 (7)	−0.0198 (7)
C21C	0.0123 (7)	0.0190 (7)	0.0190 (7)	0.0016 (5)	−0.0011 (5)	−0.0018 (5)
C22C	0.0123 (7)	0.0183 (7)	0.0191 (7)	0.0003 (5)	0.0005 (5)	−0.0027 (5)
C23C	0.0116 (6)	0.0167 (7)	0.0156 (7)	−0.0004 (5)	−0.0006 (5)	0.0003 (5)
C28C	0.0144 (7)	0.0148 (7)	0.0185 (7)	0.0010 (5)	0.0009 (5)	−0.0014 (5)
C27C	0.0158 (7)	0.0144 (6)	0.0172 (7)	−0.0026 (5)	−0.0010 (5)	0.0000 (5)
C26C	0.0122 (6)	0.0165 (7)	0.0186 (7)	−0.0010 (5)	−0.0019 (5)	0.0015 (5)
C25C	0.0126 (7)	0.0180 (7)	0.0177 (7)	0.0026 (5)	0.0015 (5)	0.0007 (5)
C24C	0.0157 (7)	0.0175 (7)	0.0163 (7)	0.0003 (5)	−0.0013 (5)	−0.0039 (5)
C30C	0.0208 (8)	0.0255 (8)	0.0283 (8)	−0.0050 (6)	−0.0059 (6)	−0.0035 (7)
C29C	0.0222 (8)	0.0270 (8)	0.0283 (8)	0.0052 (6)	0.0037 (6)	−0.0075 (7)
C32C	0.0295 (9)	0.0237 (8)	0.0303 (9)	−0.0030 (7)	0.0044 (7)	−0.0103 (7)
C31C	0.0238 (8)	0.0255 (8)	0.0331 (9)	0.0055 (6)	0.0028 (7)	−0.0094 (7)
O1A	0.0116 (5)	0.0341 (6)	0.0274 (6)	0.0058 (4)	0.0015 (4)	−0.0023 (5)
O2A	0.0170 (5)	0.0343 (7)	0.0279 (6)	−0.0068 (5)	−0.0009 (5)	−0.0123 (5)
O3A	0.0144 (5)	0.0316 (6)	0.0292 (6)	0.0032 (4)	−0.0014 (4)	−0.0045 (5)
O4A	0.0190 (6)	0.0215 (6)	0.0348 (6)	−0.0035 (4)	−0.0040 (5)	−0.0049 (5)
O1B	0.0161 (5)	0.0285 (6)	0.0319 (6)	−0.0047 (4)	0.0011 (5)	−0.0021 (5)
O2B	0.0210 (6)	0.0373 (7)	0.0302 (6)	0.0082 (5)	−0.0005 (5)	0.0110 (5)
O3B	0.0167 (5)	0.0303 (6)	0.0311 (6)	−0.0063 (5)	0.0032 (5)	0.0036 (5)
O4B	0.0161 (5)	0.0275 (6)	0.0251 (6)	0.0033 (4)	0.0003 (4)	0.0079 (5)
O2C	0.0163 (5)	0.0216 (5)	0.0223 (5)	−0.0026 (4)	−0.0044 (4)	−0.0054 (4)
O1C	0.0131 (5)	0.0258 (6)	0.0298 (6)	0.0046 (4)	0.0013 (4)	−0.0067 (5)
O4C	0.0198 (5)	0.0200 (5)	0.0271 (6)	−0.0010 (4)	−0.0044 (4)	−0.0089 (4)
O3C	0.0152 (5)	0.0270 (6)	0.0279 (6)	0.0066 (4)	−0.0012 (4)	−0.0083 (5)

### Geometric parameters (Å, °)

**Table:**

C1A—C6A	1.387 (2)	C17B—C18B	1.392 (2)
C1A—C2A	1.401 (2)	C17B—H17B	0.9500
C1A—C7A	1.4627 (19)	C18B—C19B	1.398 (2)
C2A—C3A	1.3817 (19)	C18B—C21B	1.4647 (19)
C2A—H2A	0.9500	C19B—C20B	1.3809 (19)
C3A—O1A	1.3607 (18)	C19B—H19B	0.9500
C3A—C4A	1.397 (2)	C20B—H20B	0.9500
C4A—C5A	1.379 (2)	C21B—C22B	1.329 (2)
C4A—H4A	0.9500	C21B—H21B	0.9500
C5A—O2A	1.3603 (18)	C22B—C23B	1.4650 (19)
C5A—C6A	1.3933 (19)	C22B—H22B	0.9500
C6A—H6A	0.9500	C23B—C28B	1.383 (2)
C7A—C8A	1.333 (2)	C23B—C24B	1.402 (2)
C7A—H7A	0.9500	C24B—C25B	1.383 (2)
C8A—C9A	1.4638 (18)	C24B—H24B	0.9500
C8A—H8A	0.9500	C25B—O3B	1.3605 (18)
C9A—C14A	1.391 (2)	C25B—C26B	1.397 (2)
C9A—C10A	1.398 (2)	C26B—C27B	1.379 (2)
C10A—C11A	1.3774 (19)	C26B—H26B	0.9500
C10A—H10A	0.9500	C27B—O4B	1.3597 (18)
C11A—C12A	1.3940 (19)	C27B—C28B	1.3915 (19)
C11A—H11A	0.9500	C28B—H28B	0.9500
C12A—C13A	1.392 (2)	C30B—O2B	1.4184 (19)
C12A—C15A	1.4768 (18)	C30B—H30D	0.9800
C13A—C14A	1.3876 (19)	C30B—H30E	0.9800
C13A—H13A	0.9500	C30B—H30F	0.9800
C14A—H14A	0.9500	C31B—O3B	1.419 (2)
C15A—C20A	1.3943 (19)	C31B—H31D	0.9800
C15A—C16A	1.396 (2)	C31B—H31E	0.9800
C16A—C17A	1.383 (2)	C31B—H31F	0.9800
C16A—H16A	0.9500	C32B—O4B	1.4224 (18)
C17A—C18A	1.394 (2)	C32B—H32D	0.9800
C17A—H17A	0.9500	C32B—H32E	0.9800
C18A—C19A	1.401 (2)	C32B—H32F	0.9800
C18A—C21A	1.4654 (19)	C6C—C1C	1.3866 (19)
C19A—C20A	1.3824 (19)	C6C—C5C	1.3957 (19)
C19A—H19A	0.9500	C6C—H6C	0.9500
C20A—H20A	0.9500	C5C—O2C	1.3614 (17)
C21A—C22A	1.329 (2)	C5C—C4C	1.381 (2)
C21A—H21A	0.9500	C4C—C3C	1.397 (2)
C22A—C23A	1.4649 (19)	C4C—H4C	0.9500
C22A—H22A	0.9500	C3C—O1C	1.3613 (17)
C23A—C24A	1.389 (2)	C3C—C2C	1.3813 (19)
C23A—C28A	1.400 (2)	C2C—C1C	1.405 (2)
C24A—C25A	1.393 (2)	C2C—H2C	0.9500
C24A—H24A	0.9500	C1C—C7C	1.4651 (18)
C25A—O3A	1.3559 (18)	C7C—C8C	1.329 (2)
C25A—C26A	1.381 (2)	C7C—H7C	0.9500
C26A—C27A	1.391 (2)	C8C—C9C	1.4637 (19)
C26A—H26A	0.9500	C8C—H8C	0.9500
C27A—O4A	1.3615 (18)	C9C—C14C	1.390 (2)
C27A—C28A	1.3835 (19)	C9C—C10C	1.392 (2)
C28A—H28A	0.9500	C14C—C13C	1.382 (2)
C29A—O1A	1.421 (2)	C14C—H14C	0.9500
C29A—H29A	0.9800	C13C—C12C	1.392 (2)
C29A—H29B	0.9800	C13C—H13C	0.9500
C29A—H29C	0.9800	C12C—C11C	1.3877 (19)
C30A—O2A	1.4251 (18)	C12C—C15C	1.4766 (19)
C30A—H30A	0.9800	C11C—C10C	1.377 (2)
C30A—H30B	0.9800	C11C—H11C	0.9500
C30A—H30C	0.9800	C10C—H10C	0.9500
C31A—O3A	1.4239 (18)	C15C—C16C	1.389 (2)
C31A—H31A	0.9800	C15C—C20C	1.393 (2)
C31A—H31B	0.9800	C20C—C19C	1.377 (2)
C31A—H31C	0.9800	C20C—H20C	0.9500
C32A—O4A	1.417 (2)	C19C—C18C	1.393 (2)
C32A—H32A	0.9800	C19C—H19C	0.9500
C32A—H32B	0.9800	C18C—C17C	1.388 (2)
C32A—H32C	0.9800	C18C—C21C	1.4609 (19)
C29B—O1B	1.4191 (18)	C17C—C16C	1.384 (2)
C29B—H29D	0.9800	C17C—H17C	0.9500
C29B—H29E	0.9800	C16C—H16C	0.9500
C29B—H29F	0.9800	C21C—C22C	1.327 (2)
C2B—C3B	1.384 (2)	C21C—H21C	0.9500
C2B—C1B	1.392 (2)	C22C—C23C	1.4634 (19)
C2B—H2B	0.9500	C22C—H22C	0.9500
C3B—O1B	1.3608 (19)	C23C—C28C	1.3906 (19)
C3B—C4B	1.399 (2)	C23C—C24C	1.395 (2)
C4B—C5B	1.382 (2)	C28C—C27C	1.3892 (19)
C4B—H4B	0.9500	C28C—H28C	0.9500
C5B—O2B	1.3719 (19)	C27C—O4C	1.3607 (17)
C5B—C6B	1.385 (2)	C27C—C26C	1.383 (2)
C6B—C1B	1.394 (2)	C26C—C25C	1.387 (2)
C6B—H6B	0.9500	C26C—H26C	0.9500
C1B—C7B	1.4638 (19)	C25C—O3C	1.3602 (17)
C7B—C8B	1.334 (2)	C25C—C24C	1.3880 (19)
C7B—H7B	0.9500	C24C—H24C	0.9500
C8B—C9B	1.464 (2)	C30C—O2C	1.4259 (17)
C8B—H8B	0.9500	C30C—H30G	0.9800
C9B—C14B	1.394 (2)	C30C—H30H	0.9800
C9B—C10B	1.400 (2)	C30C—H30I	0.9800
C10B—C11B	1.3825 (19)	C29C—O1C	1.4274 (19)
C10B—H10B	0.9500	C29C—H29G	0.9800
C11B—C12B	1.3980 (19)	C29C—H29H	0.9800
C11B—H11B	0.9500	C29C—H29I	0.9800
C12B—C13B	1.397 (2)	C32C—O4C	1.4278 (19)
C12B—C15B	1.4760 (19)	C32C—H32G	0.9800
C13B—C14B	1.384 (2)	C32C—H32H	0.9800
C13B—H13B	0.9500	C32C—H32I	0.9800
C14B—H14B	0.9500	C31C—O3C	1.4191 (19)
C15B—C16B	1.393 (2)	C31C—H31G	0.9800
C15B—C20B	1.395 (2)	C31C—H31H	0.9800
C16B—C17B	1.3861 (19)	C31C—H31I	0.9800
C16B—H16B	0.9500		
			
C6A—C1A—C2A	119.76 (13)	C20B—C19B—C18B	121.23 (14)
C6A—C1A—C7A	117.44 (13)	C20B—C19B—H19B	119.4
C2A—C1A—C7A	122.80 (13)	C18B—C19B—H19B	119.4
C3A—C2A—C1A	119.03 (13)	C19B—C20B—C15B	121.26 (14)
C3A—C2A—H2A	120.5	C19B—C20B—H20B	119.4
C1A—C2A—H2A	120.5	C15B—C20B—H20B	119.4
O1A—C3A—C2A	124.27 (14)	C22B—C21B—C18B	125.08 (14)
O1A—C3A—C4A	114.28 (13)	C22B—C21B—H21B	117.5
C2A—C3A—C4A	121.45 (14)	C18B—C21B—H21B	117.5
C5A—C4A—C3A	119.08 (13)	C21B—C22B—C23B	127.82 (14)
C5A—C4A—H4A	120.5	C21B—C22B—H22B	116.1
C3A—C4A—H4A	120.5	C23B—C22B—H22B	116.1
O2A—C5A—C4A	124.70 (13)	C28B—C23B—C24B	119.66 (13)
O2A—C5A—C6A	115.09 (13)	C28B—C23B—C22B	117.36 (13)
C4A—C5A—C6A	120.21 (14)	C24B—C23B—C22B	122.98 (13)
C1A—C6A—C5A	120.46 (14)	C25B—C24B—C23B	119.19 (14)
C1A—C6A—H6A	119.8	C25B—C24B—H24B	120.4
C5A—C6A—H6A	119.8	C23B—C24B—H24B	120.4
C8A—C7A—C1A	128.11 (14)	O3B—C25B—C24B	124.61 (14)
C8A—C7A—H7A	115.9	O3B—C25B—C26B	114.15 (13)
C1A—C7A—H7A	115.9	C24B—C25B—C26B	121.24 (13)
C7A—C8A—C9A	124.89 (13)	C27B—C26B—C25B	118.99 (13)
C7A—C8A—H8A	117.6	C27B—C26B—H26B	120.5
C9A—C8A—H8A	117.6	C25B—C26B—H26B	120.5
C14A—C9A—C10A	117.26 (13)	O4B—C27B—C26B	124.28 (13)
C14A—C9A—C8A	120.30 (13)	O4B—C27B—C28B	115.31 (13)
C10A—C9A—C8A	122.44 (13)	C26B—C27B—C28B	120.41 (13)
C11A—C10A—C9A	121.14 (13)	C23B—C28B—C27B	120.48 (14)
C11A—C10A—H10A	119.4	C23B—C28B—H28B	119.8
C9A—C10A—H10A	119.4	C27B—C28B—H28B	119.8
C10A—C11A—C12A	121.61 (14)	O2B—C30B—H30D	109.5
C10A—C11A—H11A	119.2	O2B—C30B—H30E	109.5
C12A—C11A—H11A	119.2	H30D—C30B—H30E	109.5
C13A—C12A—C11A	117.47 (13)	O2B—C30B—H30F	109.5
C13A—C12A—C15A	122.48 (13)	H30D—C30B—H30F	109.5
C11A—C12A—C15A	120.05 (13)	H30E—C30B—H30F	109.5
C14A—C13A—C12A	120.88 (13)	O3B—C31B—H31D	109.5
C14A—C13A—H13A	119.6	O3B—C31B—H31E	109.5
C12A—C13A—H13A	119.6	H31D—C31B—H31E	109.5
C13A—C14A—C9A	121.62 (13)	O3B—C31B—H31F	109.5
C13A—C14A—H14A	119.2	H31D—C31B—H31F	109.5
C9A—C14A—H14A	119.2	H31E—C31B—H31F	109.5
C20A—C15A—C16A	117.33 (13)	O4B—C32B—H32D	109.5
C20A—C15A—C12A	121.80 (13)	O4B—C32B—H32E	109.5
C16A—C15A—C12A	120.84 (13)	H32D—C32B—H32E	109.5
C17A—C16A—C15A	121.45 (14)	O4B—C32B—H32F	109.5
C17A—C16A—H16A	119.3	H32D—C32B—H32F	109.5
C15A—C16A—H16A	119.3	H32E—C32B—H32F	109.5
C16A—C17A—C18A	121.20 (14)	C1C—C6C—C5C	120.45 (13)
C16A—C17A—H17A	119.4	C1C—C6C—H6C	119.8
C18A—C17A—H17A	119.4	C5C—C6C—H6C	119.8
C17A—C18A—C19A	117.48 (13)	O2C—C5C—C4C	124.48 (13)
C17A—C18A—C21A	119.24 (14)	O2C—C5C—C6C	115.20 (13)
C19A—C18A—C21A	123.25 (13)	C4C—C5C—C6C	120.31 (13)
C20A—C19A—C18A	121.08 (13)	C5C—C4C—C3C	118.99 (13)
C20A—C19A—H19A	119.5	C5C—C4C—H4C	120.5
C18A—C19A—H19A	119.5	C3C—C4C—H4C	120.5
C19A—C20A—C15A	121.44 (13)	O1C—C3C—C2C	124.23 (13)
C19A—C20A—H20A	119.3	O1C—C3C—C4C	114.29 (12)
C15A—C20A—H20A	119.3	C2C—C3C—C4C	121.47 (13)
C22A—C21A—C18A	126.04 (14)	C3C—C2C—C1C	119.16 (13)
C22A—C21A—H21A	117.0	C3C—C2C—H2C	120.4
C18A—C21A—H21A	117.0	C1C—C2C—H2C	120.4
C21A—C22A—C23A	127.20 (14)	C6C—C1C—C2C	119.61 (13)
C21A—C22A—H22A	116.4	C6C—C1C—C7C	118.07 (13)
C23A—C22A—H22A	116.4	C2C—C1C—C7C	122.31 (13)
C24A—C23A—C28A	119.52 (13)	C8C—C7C—C1C	127.38 (13)
C24A—C23A—C22A	123.22 (13)	C8C—C7C—H7C	116.3
C28A—C23A—C22A	117.26 (13)	C1C—C7C—H7C	116.3
C23A—C24A—C25A	119.94 (14)	C7C—C8C—C9C	125.69 (13)
C23A—C24A—H24A	120.0	C7C—C8C—H8C	117.2
C25A—C24A—H24A	120.0	C9C—C8C—H8C	117.2
O3A—C25A—C26A	123.32 (13)	C14C—C9C—C10C	116.93 (13)
O3A—C25A—C24A	115.83 (13)	C14C—C9C—C8C	119.64 (13)
C26A—C25A—C24A	120.84 (14)	C10C—C9C—C8C	123.42 (13)
C25A—C26A—C27A	118.96 (13)	C13C—C14C—C9C	121.58 (14)
C25A—C26A—H26A	120.5	C13C—C14C—H14C	119.2
C27A—C26A—H26A	120.5	C9C—C14C—H14C	119.2
O4A—C27A—C28A	124.38 (14)	C14C—C13C—C12C	121.57 (14)
O4A—C27A—C26A	114.51 (13)	C14C—C13C—H13C	119.2
C28A—C27A—C26A	121.11 (14)	C12C—C13C—H13C	119.2
C27A—C28A—C23A	119.63 (14)	C11C—C12C—C13C	116.48 (13)
C27A—C28A—H28A	120.2	C11C—C12C—C15C	121.35 (13)
C23A—C28A—H28A	120.2	C13C—C12C—C15C	122.17 (13)
O1A—C29A—H29A	109.5	C10C—C11C—C12C	122.23 (14)
O1A—C29A—H29B	109.5	C10C—C11C—H11C	118.9
H29A—C29A—H29B	109.5	C12C—C11C—H11C	118.9
O1A—C29A—H29C	109.5	C11C—C10C—C9C	121.22 (13)
H29A—C29A—H29C	109.5	C11C—C10C—H10C	119.4
H29B—C29A—H29C	109.5	C9C—C10C—H10C	119.4
O2A—C30A—H30A	109.5	C16C—C15C—C20C	116.37 (13)
O2A—C30A—H30B	109.5	C16C—C15C—C12C	122.08 (13)
H30A—C30A—H30B	109.5	C20C—C15C—C12C	121.52 (13)
O2A—C30A—H30C	109.5	C19C—C20C—C15C	122.10 (14)
H30A—C30A—H30C	109.5	C19C—C20C—H20C	118.9
H30B—C30A—H30C	109.5	C15C—C20C—H20C	118.9
O3A—C31A—H31A	109.5	C20C—C19C—C18C	121.35 (14)
O3A—C31A—H31B	109.5	C20C—C19C—H19C	119.3
H31A—C31A—H31B	109.5	C18C—C19C—H19C	119.3
O3A—C31A—H31C	109.5	C17C—C18C—C19C	116.81 (13)
H31A—C31A—H31C	109.5	C17C—C18C—C21C	120.05 (13)
H31B—C31A—H31C	109.5	C19C—C18C—C21C	123.15 (13)
O4A—C32A—H32A	109.5	C16C—C17C—C18C	121.65 (15)
O4A—C32A—H32B	109.5	C16C—C17C—H17C	119.2
H32A—C32A—H32B	109.5	C18C—C17C—H17C	119.2
O4A—C32A—H32C	109.5	C17C—C16C—C15C	121.70 (14)
H32A—C32A—H32C	109.5	C17C—C16C—H16C	119.1
H32B—C32A—H32C	109.5	C15C—C16C—H16C	119.1
O1B—C29B—H29D	109.5	C22C—C21C—C18C	125.94 (14)
O1B—C29B—H29E	109.5	C22C—C21C—H21C	117.0
H29D—C29B—H29E	109.5	C18C—C21C—H21C	117.0
O1B—C29B—H29F	109.5	C21C—C22C—C23C	127.19 (14)
H29D—C29B—H29F	109.5	C21C—C22C—H22C	116.4
H29E—C29B—H29F	109.5	C23C—C22C—H22C	116.4
C3B—C2B—C1B	119.53 (14)	C28C—C23C—C24C	119.95 (13)
C3B—C2B—H2B	120.2	C28C—C23C—C22C	117.63 (13)
C1B—C2B—H2B	120.2	C24C—C23C—C22C	122.43 (13)
O1B—C3B—C2B	115.13 (14)	C27C—C28C—C23C	119.94 (13)
O1B—C3B—C4B	123.32 (13)	C27C—C28C—H28C	120.0
C2B—C3B—C4B	121.56 (14)	C23C—C28C—H28C	120.0
C5B—C4B—C3B	118.33 (13)	O4C—C27C—C26C	115.61 (12)
C5B—C4B—H4B	120.8	O4C—C27C—C28C	123.97 (13)
C3B—C4B—H4B	120.8	C26C—C27C—C28C	120.41 (13)
O2B—C5B—C4B	124.50 (14)	C27C—C26C—C25C	119.49 (13)
O2B—C5B—C6B	114.80 (14)	C27C—C26C—H26C	120.3
C4B—C5B—C6B	120.71 (14)	C25C—C26C—H26C	120.3
C5B—C6B—C1B	120.73 (14)	O3C—C25C—C26C	115.12 (12)
C5B—C6B—H6B	119.6	O3C—C25C—C24C	123.99 (13)
C1B—C6B—H6B	119.6	C26C—C25C—C24C	120.89 (13)
C2B—C1B—C6B	119.13 (13)	C25C—C24C—C23C	119.31 (13)
C2B—C1B—C7B	123.39 (14)	C25C—C24C—H24C	120.3
C6B—C1B—C7B	117.48 (14)	C23C—C24C—H24C	120.3
C8B—C7B—C1B	128.17 (14)	O2C—C30C—H30G	109.5
C8B—C7B—H7B	115.9	O2C—C30C—H30H	109.5
C1B—C7B—H7B	115.9	H30G—C30C—H30H	109.5
C7B—C8B—C9B	125.49 (14)	O2C—C30C—H30I	109.5
C7B—C8B—H8B	117.3	H30G—C30C—H30I	109.5
C9B—C8B—H8B	117.3	H30H—C30C—H30I	109.5
C14B—C9B—C10B	117.48 (13)	O1C—C29C—H29G	109.5
C14B—C9B—C8B	119.89 (13)	O1C—C29C—H29H	109.5
C10B—C9B—C8B	122.63 (13)	H29G—C29C—H29H	109.5
C11B—C10B—C9B	121.01 (13)	O1C—C29C—H29I	109.5
C11B—C10B—H10B	119.5	H29G—C29C—H29I	109.5
C9B—C10B—H10B	119.5	H29H—C29C—H29I	109.5
C10B—C11B—C12B	121.56 (13)	O4C—C32C—H32G	109.5
C10B—C11B—H11B	119.2	O4C—C32C—H32H	109.5
C12B—C11B—H11B	119.2	H32G—C32C—H32H	109.5
C13B—C12B—C11B	117.30 (13)	O4C—C32C—H32I	109.5
C13B—C12B—C15B	121.30 (13)	H32G—C32C—H32I	109.5
C11B—C12B—C15B	121.40 (13)	H32H—C32C—H32I	109.5
C14B—C13B—C12B	121.20 (14)	O3C—C31C—H31G	109.5
C14B—C13B—H13B	119.4	O3C—C31C—H31H	109.5
C12B—C13B—H13B	119.4	H31G—C31C—H31H	109.5
C13B—C14B—C9B	121.44 (14)	O3C—C31C—H31I	109.5
C13B—C14B—H14B	119.3	H31G—C31C—H31I	109.5
C9B—C14B—H14B	119.3	H31H—C31C—H31I	109.5
C16B—C15B—C20B	117.71 (13)	C3A—O1A—C29A	116.65 (12)
C16B—C15B—C12B	121.83 (13)	C5A—O2A—C30A	117.18 (13)
C20B—C15B—C12B	120.47 (13)	C25A—O3A—C31A	118.07 (12)
C17B—C16B—C15B	120.92 (14)	C27A—O4A—C32A	117.23 (12)
C17B—C16B—H16B	119.5	C3B—O1B—C29B	118.41 (13)
C15B—C16B—H16B	119.5	C5B—O2B—C30B	117.89 (13)
C16B—C17B—C18B	121.51 (14)	C25B—O3B—C31B	116.87 (12)
C16B—C17B—H17B	119.2	C27B—O4B—C32B	116.82 (12)
C18B—C17B—H17B	119.2	C5C—O2C—C30C	117.52 (12)
C17B—C18B—C19B	117.37 (13)	C3C—O1C—C29C	117.02 (12)
C17B—C18B—C21B	119.56 (13)	C27C—O4C—C32C	117.52 (12)
C19B—C18B—C21B	123.07 (13)	C25C—O3C—C31C	117.45 (12)
			
C6A—C1A—C2A—C3A	−0.5 (2)	C17B—C18B—C21B—C22B	177.60 (15)
C7A—C1A—C2A—C3A	179.58 (14)	C19B—C18B—C21B—C22B	−2.8 (2)
C1A—C2A—C3A—O1A	179.44 (14)	C18B—C21B—C22B—C23B	179.20 (14)
C1A—C2A—C3A—C4A	0.0 (2)	C21B—C22B—C23B—C28B	175.56 (15)
O1A—C3A—C4A—C5A	−178.75 (13)	C21B—C22B—C23B—C24B	−4.5 (2)
C2A—C3A—C4A—C5A	0.8 (2)	C28B—C23B—C24B—C25B	1.6 (2)
C3A—C4A—C5A—O2A	179.50 (14)	C22B—C23B—C24B—C25B	−178.41 (13)
C3A—C4A—C5A—C6A	−0.9 (2)	C23B—C24B—C25B—O3B	179.65 (14)
C2A—C1A—C6A—C5A	0.4 (2)	C23B—C24B—C25B—C26B	−0.7 (2)
C7A—C1A—C6A—C5A	−179.74 (13)	O3B—C25B—C26B—C27B	178.71 (13)
O2A—C5A—C6A—C1A	179.98 (14)	C24B—C25B—C26B—C27B	−1.0 (2)
C4A—C5A—C6A—C1A	0.4 (2)	C25B—C26B—C27B—O4B	−178.84 (13)
C6A—C1A—C7A—C8A	−173.06 (16)	C25B—C26B—C27B—C28B	1.8 (2)
C2A—C1A—C7A—C8A	6.8 (2)	C24B—C23B—C28B—C27B	−0.8 (2)
C1A—C7A—C8A—C9A	−179.23 (14)	C22B—C23B—C28B—C27B	179.17 (13)
C7A—C8A—C9A—C14A	179.00 (15)	O4B—C27B—C28B—C23B	179.68 (13)
C7A—C8A—C9A—C10A	−0.4 (2)	C26B—C27B—C28B—C23B	−0.9 (2)
C14A—C9A—C10A—C11A	0.5 (2)	C1C—C6C—C5C—O2C	−178.51 (13)
C8A—C9A—C10A—C11A	179.95 (14)	C1C—C6C—C5C—C4C	0.5 (2)
C9A—C10A—C11A—C12A	0.2 (2)	O2C—C5C—C4C—C3C	178.37 (13)
C10A—C11A—C12A—C13A	−1.0 (2)	C6C—C5C—C4C—C3C	−0.5 (2)
C10A—C11A—C12A—C15A	179.27 (14)	C5C—C4C—C3C—O1C	−179.65 (13)
C11A—C12A—C13A—C14A	1.1 (2)	C5C—C4C—C3C—C2C	0.3 (2)
C15A—C12A—C13A—C14A	−179.21 (13)	O1C—C3C—C2C—C1C	179.92 (14)
C12A—C13A—C14A—C9A	−0.4 (2)	C4C—C3C—C2C—C1C	0.0 (2)
C10A—C9A—C14A—C13A	−0.4 (2)	C5C—C6C—C1C—C2C	−0.2 (2)
C8A—C9A—C14A—C13A	−179.89 (13)	C5C—C6C—C1C—C7C	179.33 (13)
C13A—C12A—C15A—C20A	−31.9 (2)	C3C—C2C—C1C—C6C	0.0 (2)
C11A—C12A—C15A—C20A	147.87 (15)	C3C—C2C—C1C—C7C	−179.55 (13)
C13A—C12A—C15A—C16A	150.00 (15)	C6C—C1C—C7C—C8C	178.32 (15)
C11A—C12A—C15A—C16A	−30.3 (2)	C2C—C1C—C7C—C8C	−2.2 (2)
C20A—C15A—C16A—C17A	−1.5 (2)	C1C—C7C—C8C—C9C	178.72 (14)
C12A—C15A—C16A—C17A	176.78 (15)	C7C—C8C—C9C—C14C	−170.93 (16)
C15A—C16A—C17A—C18A	0.9 (3)	C7C—C8C—C9C—C10C	7.9 (2)
C16A—C17A—C18A—C19A	0.3 (2)	C10C—C9C—C14C—C13C	−0.4 (3)
C16A—C17A—C18A—C21A	178.35 (15)	C8C—C9C—C14C—C13C	178.54 (16)
C17A—C18A—C19A—C20A	−0.7 (2)	C9C—C14C—C13C—C12C	0.7 (3)
C21A—C18A—C19A—C20A	−178.74 (14)	C14C—C13C—C12C—C11C	−0.7 (3)
C18A—C19A—C20A—C15A	0.1 (2)	C14C—C13C—C12C—C15C	178.57 (16)
C16A—C15A—C20A—C19A	1.0 (2)	C13C—C12C—C11C—C10C	0.4 (2)
C12A—C15A—C20A—C19A	−177.24 (13)	C15C—C12C—C11C—C10C	−178.83 (15)
C17A—C18A—C21A—C22A	−166.43 (16)	C12C—C11C—C10C—C9C	−0.2 (3)
C19A—C18A—C21A—C22A	11.5 (2)	C14C—C9C—C10C—C11C	0.1 (2)
C18A—C21A—C22A—C23A	178.86 (14)	C8C—C9C—C10C—C11C	−178.75 (15)
C21A—C22A—C23A—C24A	−3.0 (2)	C11C—C12C—C15C—C16C	2.9 (2)
C21A—C22A—C23A—C28A	176.53 (15)	C13C—C12C—C15C—C16C	−176.30 (16)
C28A—C23A—C24A—C25A	−0.5 (2)	C11C—C12C—C15C—C20C	−175.23 (15)
C22A—C23A—C24A—C25A	179.02 (13)	C13C—C12C—C15C—C20C	5.5 (2)
C23A—C24A—C25A—O3A	179.81 (13)	C16C—C15C—C20C—C19C	−0.4 (2)
C23A—C24A—C25A—C26A	0.2 (2)	C12C—C15C—C20C—C19C	177.83 (15)
O3A—C25A—C26A—C27A	−179.18 (13)	C15C—C20C—C19C—C18C	0.0 (3)
C24A—C25A—C26A—C27A	0.5 (2)	C20C—C19C—C18C—C17C	−0.3 (3)
C25A—C26A—C27A—O4A	−179.96 (13)	C20C—C19C—C18C—C21C	179.99 (15)
C25A—C26A—C27A—C28A	−0.7 (2)	C19C—C18C—C17C—C16C	1.1 (3)
O4A—C27A—C28A—C23A	179.51 (13)	C21C—C18C—C17C—C16C	−179.18 (16)
C26A—C27A—C28A—C23A	0.3 (2)	C18C—C17C—C16C—C15C	−1.6 (3)
C24A—C23A—C28A—C27A	0.3 (2)	C20C—C15C—C16C—C17C	1.2 (3)
C22A—C23A—C28A—C27A	−179.27 (13)	C12C—C15C—C16C—C17C	−177.03 (16)
C1B—C2B—C3B—O1B	−179.80 (13)	C17C—C18C—C21C—C22C	168.93 (16)
C1B—C2B—C3B—C4B	−0.1 (2)	C19C—C18C—C21C—C22C	−11.3 (2)
O1B—C3B—C4B—C5B	179.74 (14)	C18C—C21C—C22C—C23C	179.92 (14)
C2B—C3B—C4B—C5B	0.1 (2)	C21C—C22C—C23C—C28C	−171.75 (15)
C3B—C4B—C5B—O2B	−179.20 (14)	C21C—C22C—C23C—C24C	8.0 (2)
C3B—C4B—C5B—C6B	0.5 (2)	C24C—C23C—C28C—C27C	−0.2 (2)
O2B—C5B—C6B—C1B	178.66 (14)	C22C—C23C—C28C—C27C	179.64 (13)
C4B—C5B—C6B—C1B	−1.1 (2)	C23C—C28C—C27C—O4C	−179.64 (13)
C3B—C2B—C1B—C6B	−0.4 (2)	C23C—C28C—C27C—C26C	0.1 (2)
C3B—C2B—C1B—C7B	−179.55 (13)	O4C—C27C—C26C—C25C	179.99 (13)
C5B—C6B—C1B—C2B	1.0 (2)	C28C—C27C—C26C—C25C	0.3 (2)
C5B—C6B—C1B—C7B	−179.80 (13)	C27C—C26C—C25C—O3C	179.86 (13)
C2B—C1B—C7B—C8B	−2.4 (2)	C27C—C26C—C25C—C24C	−0.5 (2)
C6B—C1B—C7B—C8B	178.46 (15)	O3C—C25C—C24C—C23C	−179.99 (14)
C1B—C7B—C8B—C9B	179.96 (14)	C26C—C25C—C24C—C23C	0.4 (2)
C7B—C8B—C9B—C14B	168.33 (16)	C28C—C23C—C24C—C25C	−0.1 (2)
C7B—C8B—C9B—C10B	−11.0 (2)	C22C—C23C—C24C—C25C	−179.86 (13)
C14B—C9B—C10B—C11B	0.8 (2)	C2A—C3A—O1A—C29A	7.8 (2)
C8B—C9B—C10B—C11B	−179.79 (14)	C4A—C3A—O1A—C29A	−172.69 (14)
C9B—C10B—C11B—C12B	−0.2 (2)	C4A—C5A—O2A—C30A	−10.2 (2)
C10B—C11B—C12B—C13B	−0.8 (2)	C6A—C5A—O2A—C30A	170.20 (15)
C10B—C11B—C12B—C15B	178.33 (13)	C26A—C25A—O3A—C31A	−3.1 (2)
C11B—C12B—C13B—C14B	1.3 (2)	C24A—C25A—O3A—C31A	177.21 (14)
C15B—C12B—C13B—C14B	−177.88 (14)	C28A—C27A—O4A—C32A	5.3 (2)
C12B—C13B—C14B—C9B	−0.7 (2)	C26A—C27A—O4A—C32A	−175.46 (14)
C10B—C9B—C14B—C13B	−0.4 (2)	C2B—C3B—O1B—C29B	−173.65 (13)
C8B—C9B—C14B—C13B	−179.78 (15)	C4B—C3B—O1B—C29B	6.7 (2)
C13B—C12B—C15B—C16B	−149.58 (15)	C4B—C5B—O2B—C30B	−8.0 (2)
C11B—C12B—C15B—C16B	31.3 (2)	C6B—C5B—O2B—C30B	172.27 (15)
C13B—C12B—C15B—C20B	29.9 (2)	C24B—C25B—O3B—C31B	−7.4 (2)
C11B—C12B—C15B—C20B	−149.26 (14)	C26B—C25B—O3B—C31B	172.87 (13)
C20B—C15B—C16B—C17B	−0.9 (2)	C26B—C27B—O4B—C32B	10.7 (2)
C12B—C15B—C16B—C17B	178.55 (13)	C28B—C27B—O4B—C32B	−169.93 (14)
C15B—C16B—C17B—C18B	0.4 (2)	C4C—C5C—O2C—C30C	5.9 (2)
C16B—C17B—C18B—C19B	0.4 (2)	C6C—C5C—O2C—C30C	−175.15 (13)
C16B—C17B—C18B—C21B	−179.96 (14)	C2C—C3C—O1C—C29C	−12.6 (2)
C17B—C18B—C19B—C20B	−0.7 (2)	C4C—C3C—O1C—C29C	167.30 (13)
C21B—C18B—C19B—C20B	179.72 (14)	C26C—C27C—O4C—C32C	172.34 (14)
C18B—C19B—C20B—C15B	0.1 (2)	C28C—C27C—O4C—C32C	−7.9 (2)
C16B—C15B—C20B—C19B	0.7 (2)	C26C—C25C—O3C—C31C	−170.26 (14)
C12B—C15B—C20B—C19B	−178.81 (14)	C24C—C25C—O3C—C31C	10.1 (2)

### Table 1 Relevant C—H···π contacts in the crystal packing of the title compound (Å, °).

**Table:**

Entry	*D*	H	*A*	H···*A*	*D*—H···*A*
1	C10A	H10A	*Cg*(*B*4)^i^	2.62	148
2	C17A	H17A	*Cg*(*B*3)^i^	2.86	150
3	C19A	H19A	*Cg*(*C*4)^ii^	2.80	143
4	C29A	H29a	*Cg*(*A*3)^iii^	2.79	147
5	C10B	H10B	*Cg*(*C*1)^iii^	2.84	148
6	C14B	H14B	*Cg*(*A*2)^iv^	2.97	147
7	C19B	H19B	*Cg*(*A*1)^iv^	2.67	149
8	C31B	H31F	*Cg*(*B*2)^iii^	2.80	148
9	C10C	H10C	*Cg*(*B*1)^iv^	2.70	148
10	C19C	H19C	*Cg*(*A*4)^v^	2.85	152
11	C29C	H29G	*Cg*(*C*3)^i^	2.76	143
12	C31C	H31G	*Cg*(*C*2)^iii^	2.78	140

Symmetry codes: (i) x+1, y, z; (ii) -x+1, y+1/2, -z+1/2; (iii) x-1, y, z; (iv) x, y, z; (v) -x+2, y-1/2, -z+1/2.

### Table 2 Relevant π–π contacts in the crystal packing of the title compound (Å, °).

The angle related to a pair of centroids is defined as the angle between the *Cg*(I)···*Cg*(J) vector and the normal to plane I. Centroids as in Table 1.

**Table:**

Entry	*Cg*(*I*)	*Cg*(*J*)	*Cg*···*Cg*	Angle
1	*Cg*(*A*1)	*Cg*(*A*1)^vi^	3.738 (1)	26.65
2	*Cg*(*A*4)	*Cg*(*A*4)^vii^	3.6454 (9)	25.18
3	*Cg*(*B*1)	*Cg*(*C*4)^viii^	3.713 (1)	25.39
4	*Cg*(*B*4)	*Cg*(*C*1)^ix^	3.697 (1)	26.83
5	*Cg*(*C*1)	*Cg*(*B*4)^x^	3.697 (1)	25.86
6	*Cg*(*C*4)	*Cg*(*B*1)^xi^	3.713 (1)	25.72

Symmetry codes: (vi) -x, -y+1, -z; (vii) -x+3, -y+1, -z+1; (viii) x+1, -y+1/2, z+1/2; (ix) x-2, -y+1/2, z-1/2; (x) x+2, -y+1/2, z+1/2; (xi) x-1, -y+1/2, z-1/2.

### Table 3 Relevant short contacts involving the methoxy groups in the crystal packing of the title compound (Å, °).

**Table:**

Entry	*D*	*X*	*A*	*X*···*A*	*D*–*X*···*A*
1	O2A	C30A	O1C^ix^	3.139 (2)	175.28 (12)
2	O3A	C31A	O4C^x^	3.090 (2)	160.33 (12)
3	C32A	H32A	O2C^vii^	2.71	120
4	C32A	H32c	O3A^vii^	2.55	142
5	C15B	C16B	O1B^iii^	3.204 (2)	108.54 (9)
6	O1B	C29B	O4A^vii^	3.171 (2)	143.57 (12)
7	O2B	C30B	O3B^x^	3.171 (2)	171.88 (12)
8	O4B	C32B	O1A^xii^	3.102 (2)	174.13 (12)
9	C31B	H31D	O2C^ix^	2.68	139
10	O2C	C30C	O3C^x^	3.152 (2)	161.25 (11)
11	C29C	H29H	O4B^x^	2.67	141
12	C31C	H31I	O2B^xi^	2.70	143
13	C32C	H32G	O1B^xi^	2.40	144
14	C32C	H32I	O4C^xiii^	2.69	124

Symmetry codes: (xii) -x-1, -y+1, -z; (xiii) -x, -y, -z.
